# Whole-Genome DNA Methylation Dynamics of Sheep Preimplantation Embryo Investigated by Single-Cell DNA Methylome Sequencing

**DOI:** 10.3389/fgene.2021.753144

**Published:** 2021-12-23

**Authors:** Zijing Zhang, Jiawei Xu, Shijie Lyu, Xiaoling Xin, Qiaoting Shi, Yongzhen Huang, Xiang Yu, Xiaoting Zhu, Zhiming Li, Xianwei Wang, Limin Lang, Zhaoxue Xu, Eryao Wang

**Affiliations:** ^1^ Institute of Animal Husbandry and Veterinary Science, Henan Academy of Agricultural Sciences, Zhengzhou, China; ^2^ College of Animal Science and Technology, Northwest A & F University, Yangling, China; ^3^ Animal Health Supervision Institute of Henan Province, Zhengzhou, China; ^4^ Henan Provincial Animal Husbandry General Station, Zhengzhou, China

**Keywords:** sheep, early embryo development, methylation, developmental block, single-cell DNA methylome sequencing

## Abstract

The early stages of mammalian embryonic development involve the participation and cooperation of numerous complex processes, including nutritional, genetic, and epigenetic mechanisms. However, in embryos cultured *in vitro*, a developmental block occurs that affects embryo development and the efficiency of culture. Although the block period is reported to involve the transcriptional repression of maternal genes and transcriptional activation of zygotic genes, how epigenetic factors regulate developmental block is still unclear. In this study, we systematically analyzed whole-genome methylation levels during five stages of sheep oocyte and preimplantation embryo development using single-cell level whole genome bisulphite sequencing (SC-WGBS) technology. Then, we examined several million CpG sites in individual cells at each evaluated developmental stage to identify the methylation changes that take place during the development of sheep preimplantation embryos. Our results showed that two strong waves of methylation changes occurred, namely, demethylation at the 8-cell to 16-cell stage and methylation at the 16-cell to 32-cell stage. Analysis of DNA methylation patterns in different functional regions revealed a stable hypermethylation status in 3′UTRs and gene bodies; however, significant differences were observed in intergenic and promoter regions at different developmental stages. Changes in methylation at different stages of preimplantation embryo development were also compared to investigate the molecular mechanisms involved in sheep embryo development at the methylation level. In conclusion, we report a detailed analysis of the DNA methylation dynamics during the development of sheep preimplantation embryos. Our results provide an explanation for the complex regulatory mechanisms underlying the embryo developmental block based on changes in DNA methylation levels.

## Introduction

Early sheep embryonic development involves a complex and spatiotemporal preimplantation process, starting from the zygote and lasting until the blastocyst is formed (Calarco and McLaren, 1976). This developmental process is regulated by genetic factors, the maternal environment, and maternal nutrition (Reik et al., 2001; Ashworth et al., 2009; Chason et al., 2011). The Study investigating early sheep embryo development has greatly aided the artificial culture and transplantation of sheep embryos *in vitro* (Zabihi et al., 2019), and helped accelerate the breeding process. However, *in vitro*, sheep embryo development undergoes a developmental block stage that can markedly affect embryo culture (Meirelles et al., 2004), and early sheep embryos do not survive beyond the 8-cell stage when grown under artificial conditions. During that time, the embryonic quality was declined and embryonic development was slowed. *In vitro*, inducers must be added to the culture medium to overcome the developmental block. Although it has been reported that the block period involves the transcriptional repression of maternal genes and transcriptional activation of zygotic genes (Walser and Lipshitz, 2011; Wang et al., 2019), how epigenetic factors regulate developmental block is still unclear.

DNA methylation is a crucial epigenetic mechanism influencing mammalian embryonic development (Adrian, 2002; Smith and Alexander, 2013), and has been shown to induce transcriptional silencing, genomic imprinting, and X chromosome inactivation (Li et al., 1993; Asaf and Andrew, 2007; Ryan et al., 2009; Yamanaka et al., 2010). It is found that the whole-genome DNA methylation reprogramming firstly occurs from the primordial gametes to parental embryo developmental stage, then another process happens since the preimplantation development to new generation’s embryos (Wang et al., 2019). Previously, in early embryo development, DNA methylation changes have been elucidated. Such as significant opening in chromatin occur during oocyte growth accompanied by transcriptome changes and increased DNA methylation levels (Gu et al., 2019) and Stella protein has a function in maintain of methylation in this process which would inhibit the early embryonic delay and fragmentation (Han et al., 2018); it is shown that happens strongly methylation after high global demethylation in reprogramming during the stage of human embryo preimplantation development (Zhu et al., 2017). In sheep, it also found the DNA methylation level dynamic changes *in vivo* or *vitro* sheep embryos development stage after fertilization by quantification of 5 mC immunostaining (Young and Beaujean, 2004). However, due to the limitations of the technical method, only the fuzzy methylation trend is discovered. Consequently, detailed analysis is required to reveal the quantitative and specific changes that occur in global methylation levels in sheep preimplantation embryos, and whether key genes or regions undergo methylation reprogramming during sheep embryo development.

Recent technological advances have enabled a detailed analysis of epigenetic mechanisms on a genome-wide scale (Nagano et al., 2013; Macaulay et al., 2015; Hou et al., 2016). In this study, based on current techniques, we investigated whole-genome DNA methylation levels by single-cell bisulfite sequencing (scBS-seq) in sheep preimplantation embryos at different stages of development. DNA methylation sequencing at single-cell resolution has recently been reported for early human and mouse embryos, and has the advantage of avoiding interference from aneuploid embryos that may be present in samples (Zhu et al., 2017). Here, we mapped the dynamic methylation profile during early sheep embryo development by sc-WGBS sequencing and interpreted the early embryonic developmental block based on changes in DNA methylation levels. We aimed to identify regions with significantly different methylation levels for a better understanding of sheep embryo development.

## Materials and Methods

### Sheep Embryo Collection

Samples for this study were obtained from adult (12 mouths old) female Small-tailed Han sheep (n = 3, Henan Qiming Food Co., Ltd., Ruzhou City, Henan Province). Frozen sheep sperm from a Small-tailed Han sheep with proven fertility were thawed. The semen was transferred to H-M199 solution and centrifuged at 600 rpm for 6 min to wash 3 times, and then washed twice with H-M199 solution containing 0.3% BSA, and finally the centrifuged sperm was diluted for use. Cumulus–oocyte complexes (COCs) were collected from healthy sheep ovary tissues (n = 3) using a cutting method. Sheep were euthanized by captive bolt stunning and exsanguination following the rules of animal welfare. The oocyte status was assessed under light microscopy. In this study, intracytoplasmic sperm injection (ICSI) was used for the fertilization of preimplantation embryos. Embryos were cultivated in Quinn’s Advantage cleavage medium (SAGE, Louisiana, United States) and 5% CO_2_, 5% O_2_, and 90% N_2_ at 38.5°C. Sheep embryos at the 8-cell stage (day 2), 16-cell stage (day 3), morula stage (day 4), and blastocyst stage (day 8) were identified by microscopy and separately collected. To eliminate the cumulus cells and the zona pellucida from COCs, 1 mg/ml hyaluronidase and Tyrode’s solution (Sigma, St. Louis, MO, United States) were used, and MII oocytes were recovered. All animal experiments in this study adhered to welfare standards and were agreed by the Faculty Animal Policy and Welfare Committee of Henan Academy of Agricultural Sciences (FAPWC-HAAS, Protocol number, HAASC1008).

### Single-Cell, Whole-Genome Bisulfite Sequencing Test

Single-cell, whole-genome bisulfite sequencing (SC-WGBS) was performed by Annoroad Gene Tech. Co., Ltd. Single-cell samples were lysed using 20 mg/ml proteinase K (19131, QIAGEN) for 1 h at 37°C. To determine the methylation levels after lysis, bisulfite conversion of single-cell, genome-wide, DNA samples was performed using the EZ DNA Methylation-Gold™ Kit (D5006, Zymo, Irvine, CA, United States). Bisulfite treatment converts non-methylated, but not methylated, cytosines to uracil (Docherty et al., 2009). The first strand was synthesized using an oligo1 primer with a biotin marker, and streptavidin magnetic beads were used for ‘fishing’. Klenow fragment (3'→5′ exo-) (50 U/μl; NEB, M0212M) was used to construct the first strand, and the subsequent steps were performed according to Smallwood et al. (2014). DNA was purified using 0.8× Agencourt Ampure XP beads and M-280 streptavidin Dynabeads (Life Technologies, Carlsbad, CA, United States). The oligo2 primer with a linker sequence for two-strand synthesis was added and the purified DNA was then used for polymerase chain reaction (PCR) amplification, following the manufacturer’s instructions. Amplicons were purified using 0.8× Agencourt Ampure XP beads. Then, automated selection of fragments between 300 and 800 bp was carried out on a Sage Science Pippin HT platform.

Library quantification was performed by Agilent 2100/LabChip GX Touch to detect the fragment length distribution of the library. After the expected concentration, we used real-time PCR to test the valid concentration (>10 nmol/L) to ensure library quality. Single-cell libraries were tested, and the sequencing program was run on the Hiseq X-ten sequencing platform. High-quality clean reads were selected after quality-filtering by Trimmomatic software (Bolger et al., 2014; v.0.36). After the data were filtered, bisulfite-converted alignment method was used to align the sheep genome (Oar_v.4.0) by Bismark software (Krueger and Andrews, 2011; ver. 0.9.0). The data obtained C-base methylation information and was analyzed for downstream DNA methylation information. Using Bismark Methylation Extractor (Krueger and Andrews, 2011) and method of lambda DNA spike-in, methylation coverage for every single C (methylated) was extracted and BS conversion rate was evaluated. Data were processed and presented (visualization) using R scripts.

### Data Analysis

#### Analysis of Whole-Genome Methylation Levels

To facilitate the comparison of methylation levels and correlation across samples, we applied the consecutive genomic window method to bin the sheep genome. Genome-wide methylation levels of different cell samples were calculated using all methylated CpG sites to compare differences between different samples. Specifically, used the R package methylPipe to scan the genome with 2-Kb windows (Kishore et al., 2015), and the methylation level of the sites within a window was compared across all samples using a Kruskal–Wallis test.

The sliding-window method (10 kbp) was used to calculate the methylation level in each chromosome window (distinguishing between positive and negative strands) to show the distribution of CpG methylation levels for each chromosome.

Methylation level distribution status of functional areas and the number of CpG islands (CGIs) in different stages were analyzed to describe the DNA methylation pattern and regulatory status (Guo et al., 2013).

#### Clustering and Difference Analysis

Clustering was performed to find the CpG sites shared by the cell samples and calculate the methylation level of each point. Then, the above CpG sites were clustered according to their methylation levels. In theory, the same types of cells should present similar methylation patterns.

Differential methylation levels according to embryo developmental stage were evaluated by identifying differentially methylated regions (DMRs) using DSS software (Hao et al., 2014), and DSS detection was tested by the Wald statistic, based on beta-binomial distribution.

#### Differentially Methylated Regions Analysis

The positional distribution of DMRs on each chromosome was identified and counted. Then, the lengths of relatively hypermethylated and hypomethylated DMRs were compared between two development stages. Furthermore, based on their important biological significance, structural annotation of DMRs was performed using annotation information of gene functional elements of the reference genome, and the genes that intersected with DMRs were deemed DMR-related genes.

#### Enrichment Analysis

Gene Ontology (GO) functional enrichment analysis was performed for genes where the DMR overlapped with the promoter and gene body based on the annotation results of the DMR on the genome. The GO analysis was carried out using the DAVID online tool (http://david.abcc.ncifcrf.gov/) (Huang et al., 2009).

GO terms with an adjusted *p*-value of less than 0.05 were considered to be significantly enriched in the differentially methylated gene.

The cell signaling pathway analysis was enriched the DMR-related genes to some significance pathways by KEGG Pathway software (Kanehisa et al., 2016). Some significantly associated pathways were evaluated with a *q*-value (*q* < 0.05, adjusted using the Benjamini-Hochberg [BH] method) calculated by the hypergeometric test.

## Results

### Global Methylation Dynamics of the Genome During Sheep Preimplantation Development

**FIGURE 1:**
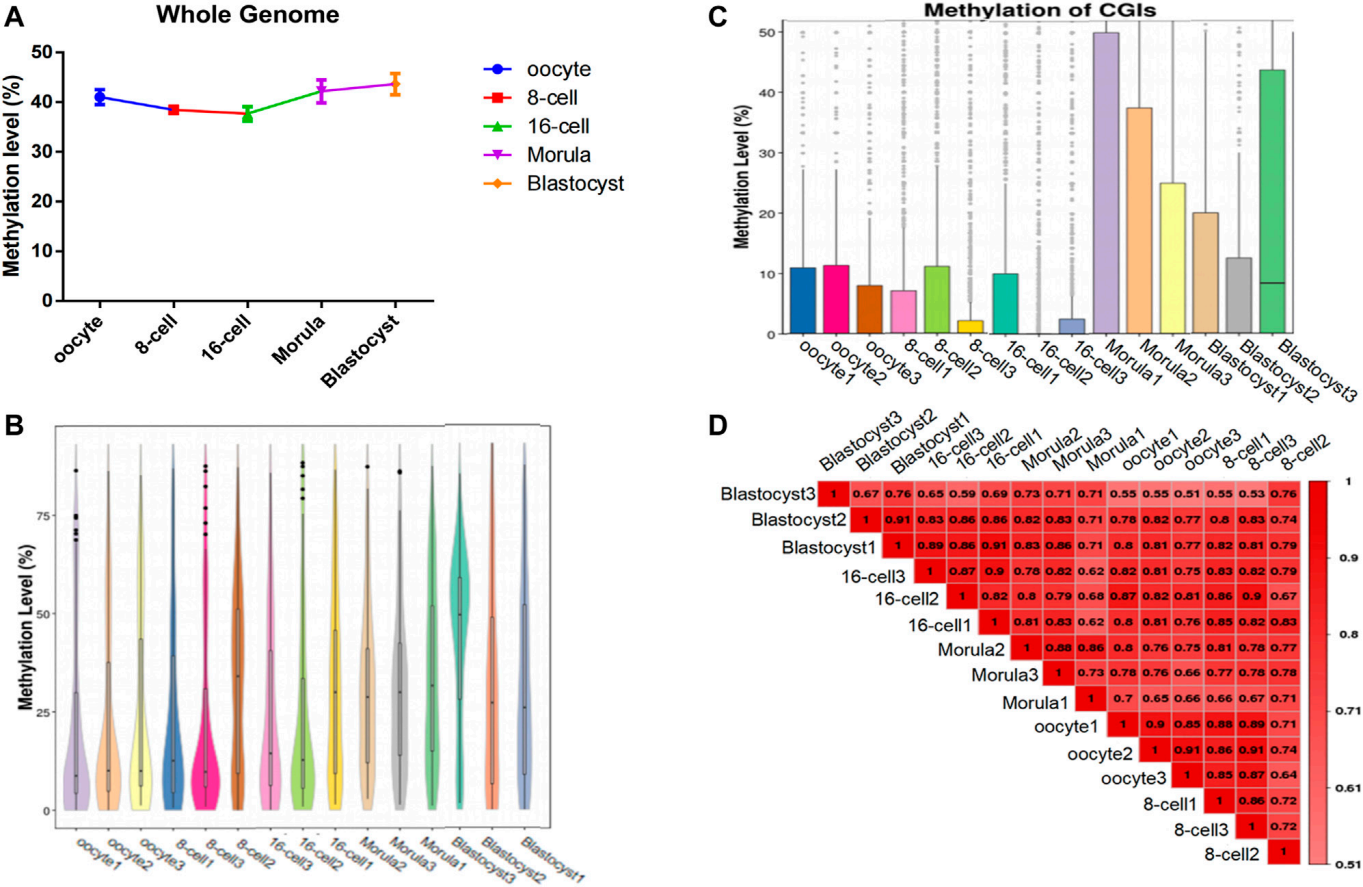
Genome-wide methylation dynamics during the development of sheep preimplantation embryos. **(A)** Whole-genome DNA methylation map in each oocyte/embryo. The horizontal axis represents different cells and the vertical axis represents the genome-wide methylation level of different cells. **(B)** Whole-gene window methylation map in each oocyte/embryo. Violin plot of the genome-wide distribution of methylation levels of whole-gene 2-kbp windows. The horizontal axis represents different cells; the vertical axis represents the level of methylation of the whole-genome window of different cells. **(C)** Methylation level distribution of CpG islands (CGIs) in each oocyte/embryo. The horizontal axis represents the cell sample and the vertical axis represents the methylation level of different cellular CGI regions. **(D)** The methylation parttern correlation analysis in sheep oocytes and early embryos. The genome-wide methylation level was calculated using the sliding window method (2 kbp), and Pearson’s correlation coefficient of all the samples was calculated. The closer the correlation coefficient is to 1, the higher the similarity of the whole-gene methylation pattern between samples.

To analyze the genome-wide changes in methylation levels during different stages of sheep preimplantation embryo development, we sequenced single cell (n = 3) from the mature oocyte to the blastocyst stage (3 × 5 stages = 15 cells) using scWGBS (Supplementary Table S1). After mapping these reads to the sheep genome, we obtained an average of 72,235,262 mapped reads with a total of 132,905,065 methylated sites (≥1×) (Supplementary Tables S2, S3). Detailed analysis indicated that the DNA methylation level decreased continuously from the oocyte to the 16-cell stage, and then increased from the 16-cell stage to the blastocyst stage (Figures 1A,B). Furthermore, the CpG island (CGI) methylation level showed a similar trend, and presented the lowest methylation level at the 16-cell stage (Figure 1C). Pair-wise Pearson’s correlation was used to compare differences in methylation levels between groups (Figure 1D). With the embryo development, the difference methylation pattern shown in different stages and differences was showed the increasing trend.

**FIGURE 2:**
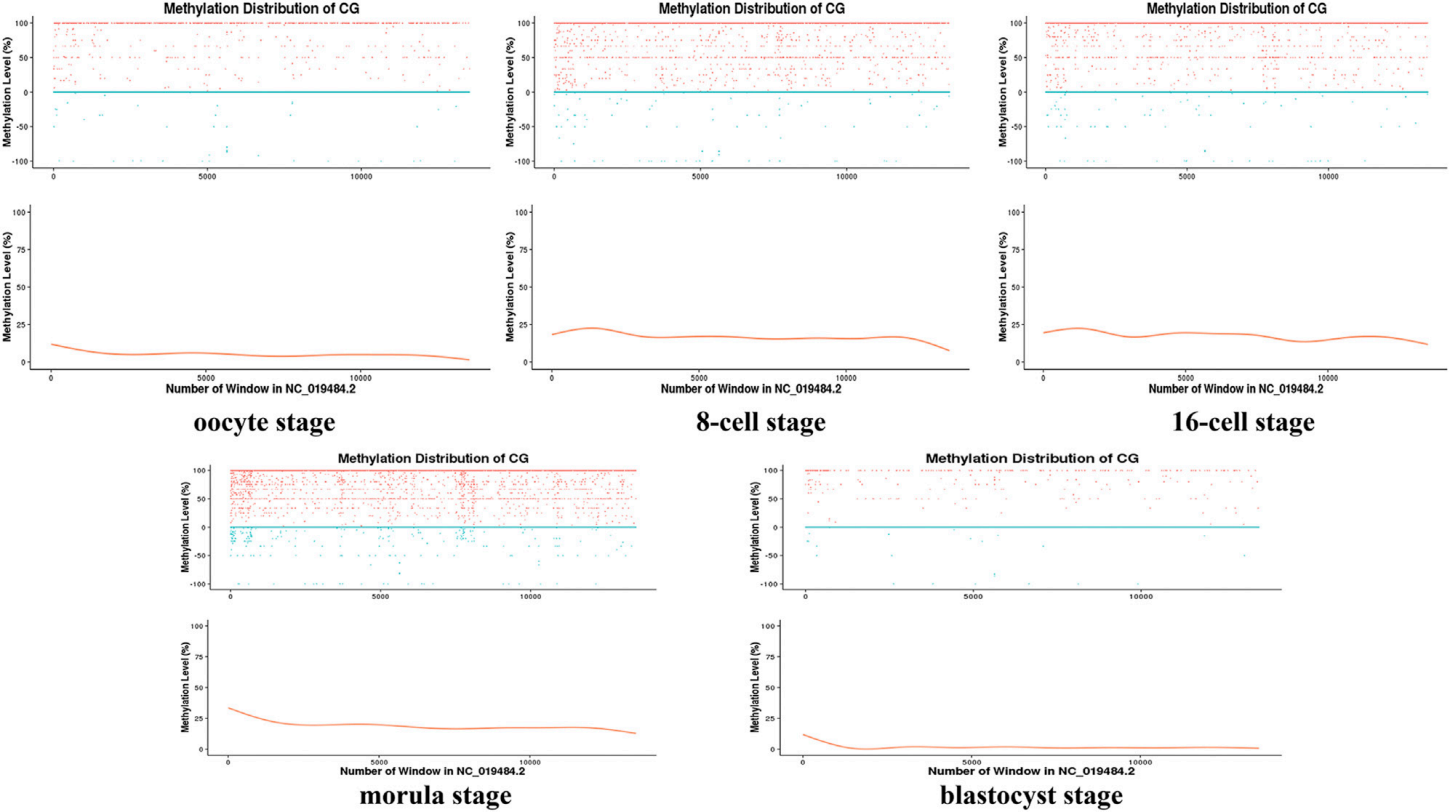
Distribution map of the methylation level of the X chromosome at different stages. The sliding window method (10 kbp) was used to calculate the level of methylation in each chromosome window (distinguishing between positive and negative strands) to delineate the methylation level pattern on a single chromosome. The horizontal axis represents the window number (i.e., the length of the chromosome) in the X chromosome (NC_019484.2); for the vertical axis, the upper part is the methylation level of each window on the positive and negative strands (scatter plot), and the lower part is the level of methylation (curve) that does not distinguish between the positive and negative strands.

Moreover, during the development of the early sheep embryo, individual chromosomes also presented different methylation levels according to developmental stage. For example, the X chromosome had a low methylation level in the oocyte, which increased at the 8-cell and 16-cell stages, and then decreased again from the morula to the blastocyst stage (Figure 2). DMR analysis showed that DMRs highly enriched to the 5′ start region of the X chromosome, and had a low level in the 3′ end region (Figures 4C, Figure 5C and Figure 6C).

### Functional Region Methylation Annotation and Cluster Analysis of Differential Sites

**FIGURE 3:**
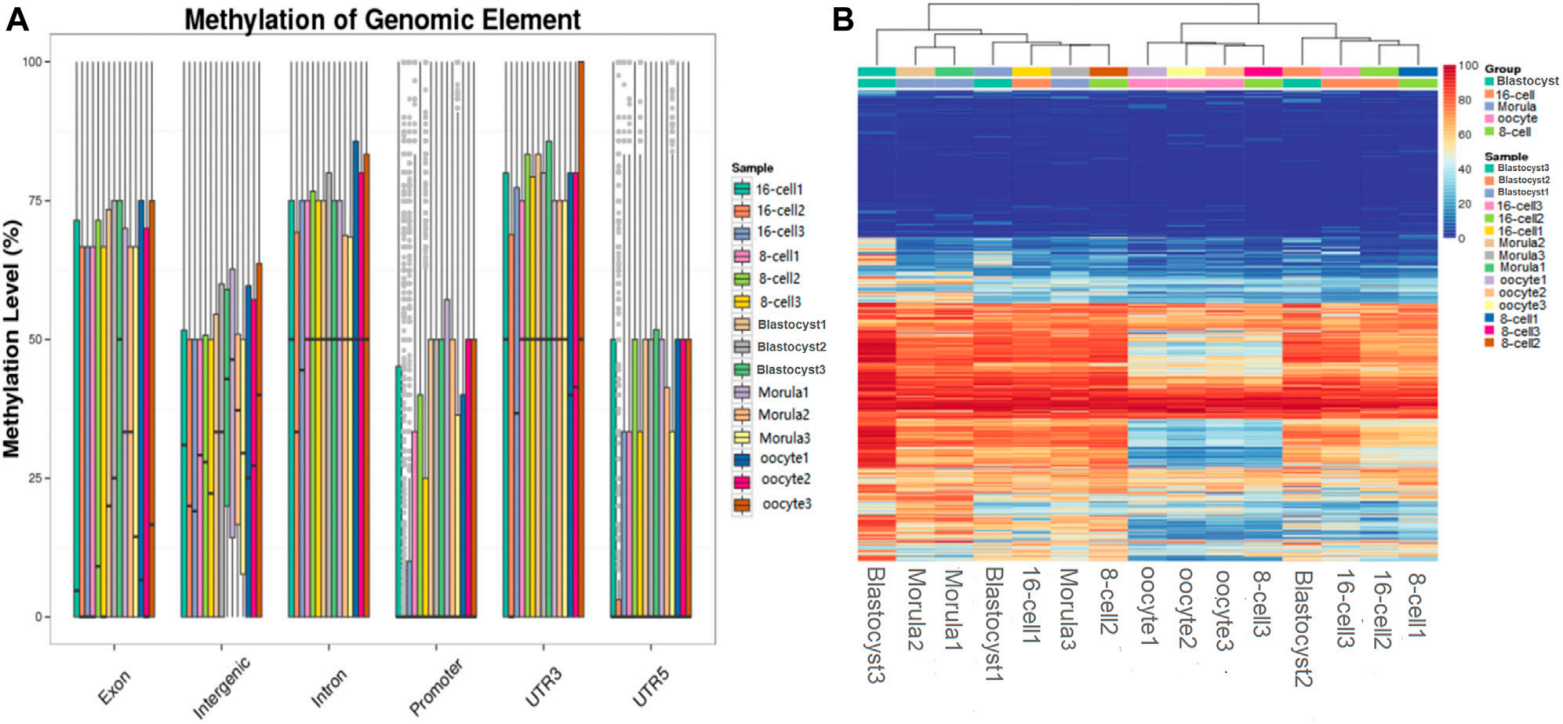
Functional region methylation annotation and cluster analysis of differential sites. **(A)** Methylation level distribution in different genome functional regions. The horizontal axis represents different functional regions in the genome; the vertical axis represents the methylation level of different samples. **(B)** Clustering map of the CpG site methylation level. The CpG sites shared by the cell samples were searched and the methylation levels of each site were calculated. Subsequently, the CpG sites were clustered based on the methylation level, and the same type of cells were clustered together to have a similar methylation pattern. Each row represents a CpG site, and each column represents a cell sample and the right scale value is the methylation level (0–100).

Analysis of DNA methylation patterns in different functional regions revealed a stable hypermethylation status in the 3′UTRs and gene bodies; however, significant differences were detected in the intergenic and promoter regions at different developmental stages. In the promoter regions, a methylation pattern similar to that of the whole genome was observed. The methylation level was downregulated until the 16-cell stage, returned to a high level until the morula stage, and then remained stable (Figure 3A).

Cluster analysis of differential CpG sites indicated that the methylation level of several maternal genome methylation sites increased during embryo development. The methylation status remained low at a large number of sites during all the stages of preimplantation embryo development. In contrast, some CpG sites remained highly methylated during these stages. Interestingly, in the morula, sudden changes in methylation levels were detected (sudden hypomethylation or hypermethylation), which then returned to their original level (Figure 3B).

### Methylation Analysis in the Developmental Block Stage

**FIGURE 4:**
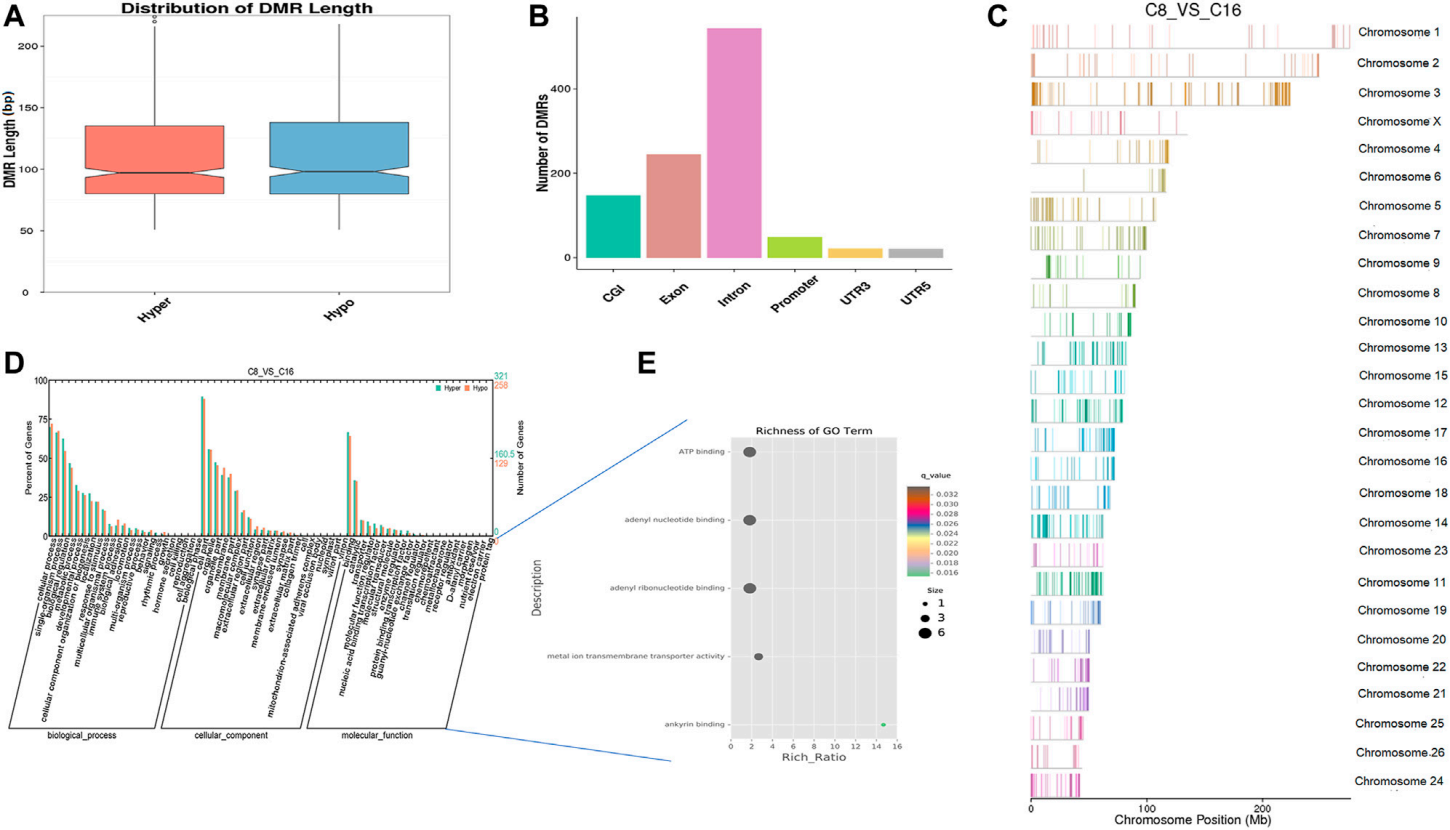
Differentially methylated analysis from the 8 to the 16-cell stage. **(A–C)** DMR length, functional annotation, and distribution in chromosomes at these stages. The length of DMRs: the horizontal axis represents the methylation status of the DMRs, Hyper is relatively hypermethylated, Hypo is relatively hypomethylated, and the vertical axis represents the length of the DMRs **(A)**. The functional annotation of DMRs: the horizontal axis represents different genomic functional regions, promoters, exons, etc.; the vertical axis represents the number of DMRs annotated to a particular functional region **(B)**. The location of DMRs in the sheep genome: the horizontal axis represents the chromosomal location, the vertical axis represents the chromosome **(C)**. **(D, E)** GO enrichment analysis. Based on the structural annotation results of DMRs on the genome, functional enrichment analysis was performed for genes where the DMR overlapped with the promoter and the gene body. The abscissa is each major class under GO terms, the left ordinate is the proportion of the class, and the right ordinate is the specific number of genes in the class. Different colors represent different groups **(D)**. The significantly enriched GO term was screened for scatter plot display. The size of each dot indicates the number of differential methylated genes contained in the term, and the color of the dots corresponds to a different *q*-value. Rich Ratio refers to the ratio between the number of differentially methylated genes enriched in the GO term and the background number. The larger the Rich Ratio, the greater the degree of enrichment. The *q*-value is the adjusted *p*-value after multiple hypothesis testing. The *q*-value is (0,1). The smaller the *q*-value, the more significant the enrichment **(E)**.

*In vitro*, early sheep embryos stop their development and do not survive beyond the 8-cell stage, which is known as the developmental block. An *in vitro* environment similar to that found in the maternal body is needed to break the block. Based on previous studies, we compared the genome methylation status in 8-cell and 16-cell stage embryos by DMR analysis. We identified 1,036 DNA regions corresponding to DMRs (528 hyper and 508 hypomethylated regions) and the lengths of the DMRs ranged from 50 bp to 4 kbp (Supplementary Table S4). The length distribution of DMRs concentrated on 50 to 150 bp (Figure 4A), and DMRs were mostly located in introns (Figure 4B). In the hypomethylated regions, the intron in LOC105616478 contained a 4.3-kbp-long differentially methylated region enriched in methylation information, while a 3.8-kbp-long intergenic region in chromosome 10 contained more hypermethylated information than other DMRs. Analysis of DMR data showed that the whole-genome methylation level was lower at the 16-cell stage than at the 8-cell stage. GO analysis showed that the set of genes where the DMR overlapped with the promoter and gene body was enriched for terms associated with cellular process, development, and transporter, indicative of transcriptional repression of maternal genes and transcriptional activation of zygotic genes during the transitional phase, potentially due to DNA methylation changes. DMR-related genes were significantly enriched for terms associated with ATP binding, adenyl ribonucleotide/nucleotide binding, and metal ion transporter activity (*q* < 0.05) (Figures 4D,E, Supplementary Tables S5, S6). Furthermore, KEGG pathway analysis indicated that the DMRs were significantly enriched for genes associated with 33 signaling pathways (*q* < 0.05), such as MAPK, PI3K/AKT, Wnt, and GnRH pathways (Supplementary Table S7).

### Methylation Analysis From the 16-Cell Stage to the Blastocyst Stage

**FIGURE 5:**
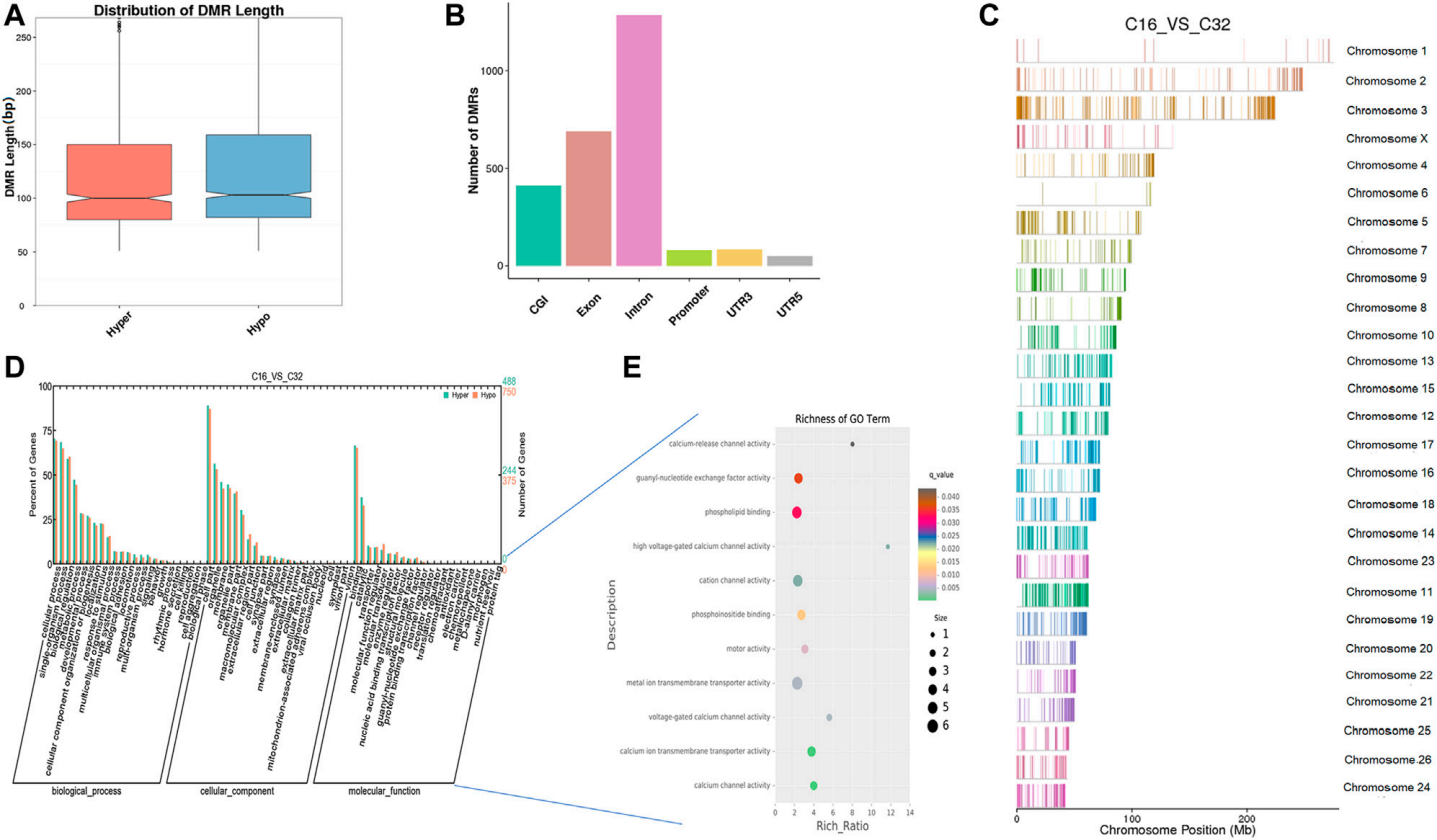
Differentially methylated analysis from the 16-cell stage to the morula stage. **(A–C)** DMR length, functional annotation, and distribution in chromosomes at these stages. The length of DMRs: the horizontal axis represents the methylation status of the DMRs, Hyper is relatively hypermethylated, Hypo is relatively hypomethylated, and the vertical axis represents the length of the DMRs **(A)**. The functional annotation of DMRs: the horizontal axis represents different genomic functional regions, promoters, exons, etc.; the vertical axis represents the number of DMRs annotated to a particular functional region **(B)**. The location of DMRs in the sheep genome: the horizontal axis represents the chromosomal location, the vertical axis represents the chromosome **(C)**. **(D, E)** GO enrichment analysis. Based on the structural annotation results of the DMRs in the genome, functional enrichment analysis was performed for genes where the DMR overlapped with the promoter and gene body. The abscissa is each major class under GO terms, the left ordinate is the proportion of the class, and the right ordinate is the specific number of genes in the class. Different colors represent different groups **(D)**. The significantly enriched GO term was screened for scatter plot display. The size of each dot indicates the number of differential methylated genes contained in the term, and the color of the dots corresponds to a different *q*-value. Rich Ratio refers to the ratio between the number of differentially methylated genes enriched in the GO term and the background number. The larger the Rich Ratio, the greater the degree of enrichment. The *q*-value is the adjusted *p*-value after multiple hypothesis testing. The *q*-value is (0,1). The smaller the *q*-value, the more significant the enrichment **(E)**.

We compared the genome methylation status among 16-cell to blastocyst stage embryos by DMR analysis. We identified 2,394 DNA regions corresponding to DMRs (865 hyper- and 1,529 hypomethylated regions) between the 16-cell stage and the morula stage and the lengths of the DMRs ranged from 50 bp to 7 kbp (Supplementary Table S4). The distribution of DMRs length also showed it was mainly 100bp and these regions were mostly located in introns (Figures 5A,B). In the hypomethylated regions, the *FXON3* gene comprised a 7-kbp differentially methylated region enriched in methylation information, while a 4.8-kbp-long region in the *COL5A1* gene contained more hypermethylated information than other DMRs. By comparing the DMRs of 16-cell and morula stage embryos, we detailed the sheep hypermethylation status at the level of the genome. GO analysis showed that this set of genes where the DMR overlapped with the promoter and gene body were enriched in terms associated with the cellular process, development, and transporter. DMR-related genes were significantly enriched in ion transporters, especially those related to calcium, such as cation channel, calcium channels, and metal ion transmembrane transporters (*q* < 0.05). Other enriched GO terms included motor activity, phosphoinositide/phospholipid binding, and guanyl-nucleotide exchange factor activity (Figures 5D,E, Supplementary Tables S5, S6). In addition, KEGG pathway analysis showed that the DMR-related genes were significantly associated with 53 signaling pathways (*q* < 0.05), such as the calcium signaling pathway, phosphatidylinositol signaling system, Wnt signaling pathway, estrogen signaling pathway, etc. (Supplementary Table S7).

**FIGURE 6:**
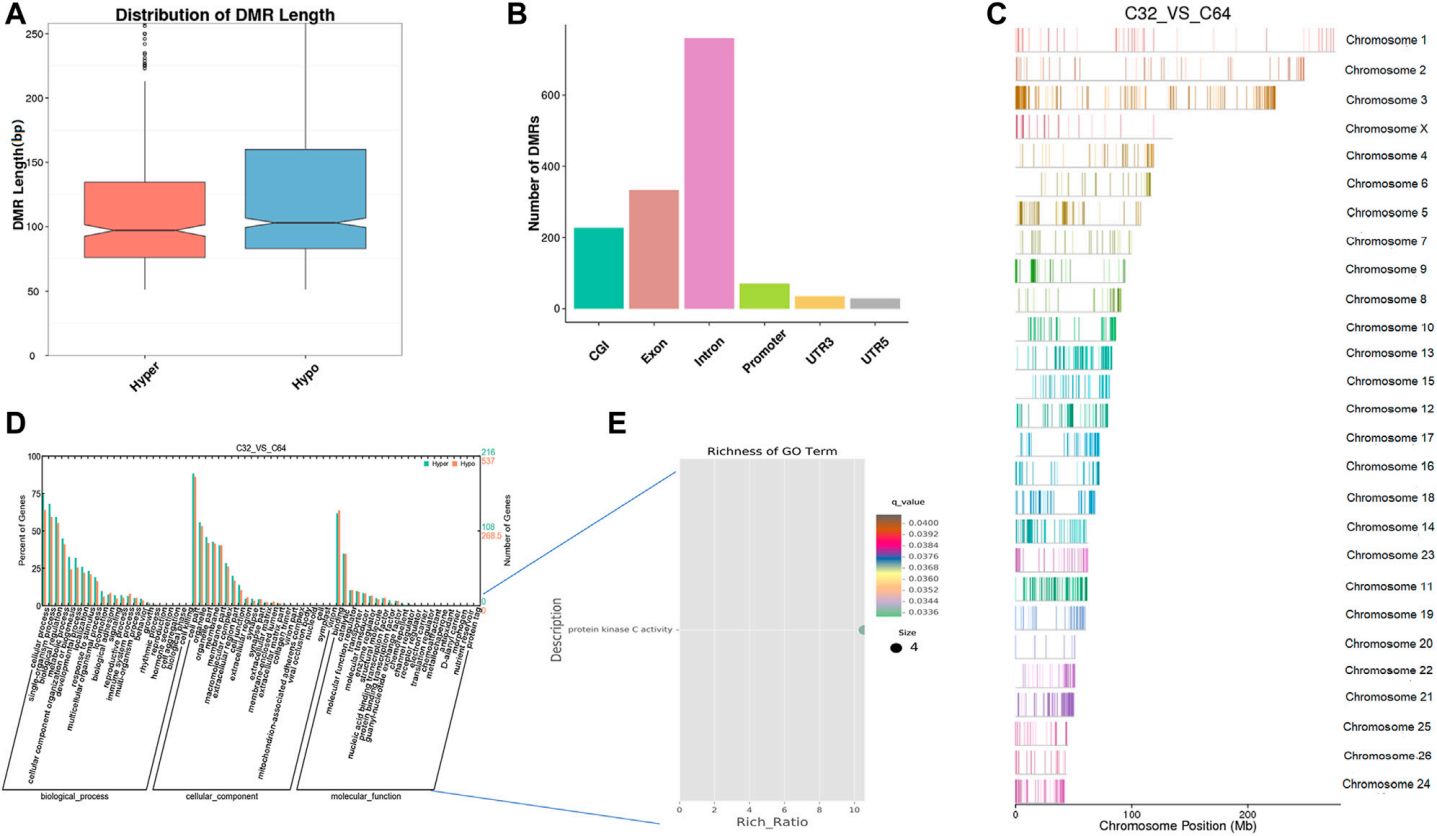
Differentially methylated analysis from the morula stage to the blastocyst stage. **(A**–**C)** DMR length, functional annotation, and distribution in chromosomes at these stages. The length of DMRs: the horizontal axis represents the methylation status of the DMRs, Hyper is relatively hypermethylated, Hypo is relatively hypomethylated, and the vertical axis represents the DMR length **(A)**. The functional annotation of DMRs: the horizontal axis represents different genomic functional regions, promoters, exons, etc.; the vertical axis represents the number of DMRs annotated to a particular functional region **(B)**. The location of DMRs in the sheep genome: the horizontal axis represents the chromosomal location, the vertical axis represents the chromosome **(C)**. **(D**, **E)** GO enrichment analysis. Based on the structural annotation results of the DMRs in the genome, functional enrichment analysis was performed for genes in which the DMR overlaps the promoter and the gene body. The abscissa is each major class under GO terms, the left ordinate is the proportion of the class, and the right ordinate is the specific number of genes in the class. Different colors represent different groups **(D)**. The significantly enriched GO term was screened for scatter plot display. The size of each dot indicates the number of differential methylated genes contained in the term, and the color of the dots corresponds to a different *q*-value. Rich Ratio refers to the ratio between the number of differentially methylated genes enriched in the GO term and the background number. The larger the Rich Ratio, the greater the degree of enrichment. The *q*-value is the adjusted *p*-value after multiple hypothesis testing. The *q*-value is (0,1). The smaller the *q*-value, the more significant the enrichment **(E)**.

Furthermore, we identified 1,499 DNA regions corresponding to DMRs (1,081 hypomethylated and 418 hypermethylated) in the morula to the blastocyst stage and these DMRs ranged from 50 bp to 8 kbp (Supplementary Table S4). According to length distribution, hypermethylated type has a wider range than hypomethylated type (Figure 6A), and both were mostly located in introns (Figure 6B, Supplementary Table S4). A detailed analysis of the DMRs showed that the *ADRBK2* gene comprised the longest region of hypomethylation, while the *NOL4L* gene contained the longest region of hypermethylation. The results also showed that the global DNA methylation level at the blastocyst stage was markedly downregulated when compared with that at the morula stage. GO analysis showed that the set of genes where the DMR overlapped with the promoter and gene body was enriched in terms associated with cellular process, development, and transporters. DMR-related genes were significantly enriched in protein kinase C activity (*q* < 0.05) (Figures 6D,E, Supplementary Tables S5, S6). Moreover, KEGG pathway analysis showed that the DMR-related genes were significantly associated with the MAPK signaling pathway, phosphatidylinositol signaling, calcium signaling pathway, circadian entrainment, inositol phosphate metabolism, retrograde endocannabinoid signaling, and cholinergic synapse (*q* < 0.05) (Supplementary Table S7).

## Discussion

The development of the sheep embryo is a complex and dynamic process that is influenced by numerous factors. During this process, the embryo phenotype changes at each stage of growth. Then, the phenotype is decided by the genetic effect and the epigenetic effect. Furthermore, due to the embryo that staying in the maternal environment, its development makes many epigenetic factors to regulate the gene expression followed by the spatiotemporal effect. Thus, the embryo culture easily happens the developmental block *in vitro* environment, whereas causing the low ratio of living in preimplantation embryo (Petters, 1992). DNA methylation is an epigenetic mechanism playing a critical role in silencing gene expression and maintaining genome stability (Jaenisch and Bird, 2003; Cedar and Bergman, 2012). Although methylation has been reported to affect early embryonic development in sheep, the detailed methylation dynamics remain unclear (Masala et al., 2017). To date, genome-wide DNA methylation patterns have been analyzed in humans through genome-wide methylation site scanning to explore how methylation affects biological development, including the directional differentiation of stem cells and regulation of embryonic gene expression (Zhang et al., 2006).

Here, we analyzed the changes in genome-wide methylation levels occurring in sheep preimplantation embryo development, from the mature oocyte to the blastocyst stage, using scWGBS-seq. Two strong waves of changes in the methylation level were identified: First, the DNA methylation level showed a continuous decrease from the oocyte to the 16-cell stage, and then increased from the 16-cell to the blastocyst stage. Our results indicate that methylation during sheep embryo development is dynamic, resulting from methylation reprogramming that occurs at the whole-genome level. (Zhu et al., 2017). Furthermore, in sheep embryo development, the 16-cell stage may represent the lowest level of methylation; additionally, a large number of zygotic genes begin to be transcribed and expressed at this stage (Ferrer et al., 1995). Comparing the changes in the methylation dynamics of cattle and human embryos before preimplantation, due to differences in species and maternal environment, their characteristics are different in developmental timing (Duan et al., 2019; Wang et al., 2019). However, they all showed a very low methylation state during the major embryonic genome activation (EGA) period (Zhu et al., 2017; Duan et al., 2019). These observations indicate that changes in methylation levels are required to release the embryo from transcriptional inhibition. In addition, big variations shown in DNA methylation (CGIs %) across the embryos (such as in 16-cell and blastocyst) at the same stage may be caused by the heterogeneity between cells (Huan et al., 2018; Luo et al., 2018), or due to differences in the development speed of the separated cells at the time of sampling although they are in the same period.

The X chromosome has many unique epigenomic features. In humans, the methylation levels of the X chromosome have been analyzed, and efforts have been undertaken to provide a unique chromosome-wide methylation profile to explain X-chromosome inactivation (XCI) (Miga et al., 2020). In the early sheep embryo, we found that the X chromosome also exhibits differential methylation patterns during early development. This indicated that the X chromosome might inactivate in early sheep embryo development stages and reactivate at the morula period. (Guo et al., 2017; Duan et al., 2019). We also described some methylation characteristics in the X chromosome of sheep. It is speculated that X chromosome methylation changes are related to chromatin conformation (Miga et al., 2020).

The distribution of methylated CpG sites in the sheep genome showed the presence of a stable hypermethylation status in 3′UTRs and gene bodies at different developmental stages. Gene-body methylation may play a pivotal role in mRNA stability and alternative splicing (Lev Maor et al., 2015). Our results showed that a stable level of gene-body methylation may maintain and regulate mRNA diversity during the development of early sheep embryos. Then, introns consistently showed higher levels of DMRs. There are some reports that the intronic DMR plays a role in the imprinting of gene (Wutz et al., 1997; Okamura et al., 2000; Smith et al., 2003). The DMR located on the intron may regulate the progress of preimplantation embryonic development by affecting the expression pattern of imprinted genes. The 3′UTR is important for regulation of gene transcription (Zlotorynski, 2015), and high levels of 3′UTR methylation may interfere with gene expression (Malumbres et al., 1999). Therefore, suppression of the exprssion of some embryonic development-related genes may be due to 3′UTR methylation.

In contrast, we found significant differences in the methylation status of CpG sites in the intergenic and promoter regions. Previous studies have suggested that DNA methylation in intergenic regions may regulate ncRNA expression and that enhancer loci in methylated intergenic regions can influence gene expression (Ehrlich et al., 2016; Hui et al., 2017). The promoter methylation status will affect the progress of transcription (Jones, 2012; Baubec and Schübeler, 2014). Promoter regions exhibited a pattern of methylation similar to that observed at the genome-wide level. Therefore, we inferred that changes in promoter methylation levels were involved in zygotic gene activation at the special stage.

Cluster analysis of differential CpG sites indicated that several maternal genome methylation sites changed from a low to a high level of methylation during embryo development. This was indicative of the transition from a maternal to zygotic control of gene expression during embryonic development. A large number of maternal proteins and mRNAs are initially synthesized and deposited in the oocyte. However, maternal mRNA and protein pools are gradually depleted, and development becomes dependent on the activity of the zygotic genome (Walser and Lipshitz 2011; Wang et al., 2019). Interestingly, we found that sudden changes in methylation levels occur at the morula stage (sudden hypo- or hypermethylation), but then return to their original levels. During the morula to blastocyst stage, the embryo starts to differentiate into a more complex form, which requires additional regulation of gene expression. Genes that showed methylation changes at this stage may have a role in the transition from the morula to the blastocyst stage.

The developmental block greatly affects embryo survival and development; however, the specific mechanisms that lead to the developmental block are still unclear, and likely involve numerous factors and regulatory mechanisms. In sheep embryos, the developmental block always occurs at the 8 to 16 cell stage. In our study, the methylation level of the sheep genome is greatly decreased at this stage. Thus, we speculate that DNA methylation may influence this block and subsequent embryo development. Several mechanisms have been proposed to explain the blocking phenomenon. The first hypothesis is that the embryo developmental block may be due to activation and occurrence of auto-apoptosis induced by *in vitro* environmental effects (Jurisicova et al., 1996; Garcia et al., 2015). BAX and BCL-2, apoptosis-related proteins, are expressed in the early embryo and are known to also have roles in developmental processes (Boumela et al., 2009; Chao et al., 2018). However, the BAX gene was not among those found to contain DMRs during early sheep embryo development, whereas the BCL2 gene presented three intronic DMRs and showed hypomethylation during the 16-cell to morula stage (Supplementary Table S4). BCL-2 is an inhibitor of apoptosis (Korsmeyer 1992; Strzyz, 2017). The result indicates that methylation may affect the expression of antiapoptotic genes in sheep embryos so as to maintain embryo development. Moreover, it has been reported that transferrin can help mouse embryos break through the 2-cell stasis phase and significantly improve embryo survival and development rates *in vitro* (Nasresfahani et al., 1990). Notably, we found that methylation changes from the 8- to 16-cell stage were significantly associated with metal ion transporter activity. From the 16-cell to the morula stage, methylation affected genes coding for ion transporters, especially calcium transporters (Supplementary Table S6). Embryonic development requires calcium ions, which are required for the activity of related ion channels and transporters (Ingrid et al., 2018; Liu et al., 2018). This finding indicates that metal ion regulation is important for early sheep embryo development.

Due to the reduced level of genome methylation at 8 to 16-cell stage, a large number of functional genes will be transcribed. Many signaling pathways that have roles in regulating embryonic development were activated, such as the PI3K/AKT, Wnt, and GnRH pathways (Supplementary Table S7). For example, the hypomethylation of receptors in the GnRH pathway will lead to the expression of FSH, which can overcome the embryo developmental block (Loutradis et al., 1995).

In conclusion, we report a detailed analysis of the DNA methylation dynamics in the development of sheep preimplantation embryos. Our results provide an explanation for the complex regulatory mechanisms underlying the embryo developmental block based on changes in the levels of DNA methylation.

## Data Availability

The datasets presented in this study can be found in NCBI, accession number: GSE190746 https://www.ncbi.nlm.nih.gov/geo/query/acc.cgi?acc=GSE190746.
